# High-resolution genomics identifies pneumococcal diversity and persistence of vaccine types in children with community-acquired pneumonia in the UK and Ireland

**DOI:** 10.1186/s12866-024-03300-w

**Published:** 2024-04-27

**Authors:** Juan Pablo Rodriguez-Ruiz, Basil Britto Xavier, Wolfgang Stöhr, Liesbet van Heirstraeten, Christine Lammens, Adam Finn, Herman Goossens, Julia Anna Bielicki, Michael Sharland, Surbhi Malhotra-Kumar, on behalf of the PERUKI, GAPRUKI and CAP-IT networks, Diana M. Gibb, Mark D. Lyttle, Sam Barratt, David Dunn, Michelle Clements, Kate Sturgeon, Elizabeth Molyneux, Chris C. Butler, Alan Smyth, Catherine Prichard, Tim E. A. Peto, Simon Cousens, Stuart Logan, Alasdair Bamford, Anna Turkova, Anna L. Goodman, Felicity Fitzgerald, Saul N. Faust, Colin Powell, Paul S. Little, Julie Robotham, Mandy Wan, Nigel Klein, Louise Rogers, Elia Vitale, Daniel B. Hawcutt, Mathew Rotheram, Stuart Hartshorn, Deepthi Jyothish, James G. Ross, Poonam Patel, Stefania Vergnano, Jeff Morgan, Godfrey Nyamugunduru, John C. Furness, Susannah J. Holt, John Gibbs, Anastasia E. Alcock, Dani Hall, Ronny Cheung, Arshid Murad, K. M. Jerman, Chris Bird, Tanya K. Z. Baron, Fleur Cantle, Niall Mullen, Rhona McCrone, Gisela Robinson, Lizzie Starkey, Sean O’Riordan, Damian Roland, Srini Bandi, Chris Gough, Sharryn Gardner, M. J. Barrett, Emily K. Walton, Akshat Kapur, Steven J. Foster, R. M. Bland, Ben Bloom, Ami Parikh, Katherine Potier, Judith Gilchrist, Noreen West, Paul T. Heath, Yasser Iqbal, Ian K. Maconochie, Maggie Nyirenda, Sophie Keers, Katrina Cathie, Jane Bayreuther, Elizabeth-Jayne L. Herrieven, Willian Townend

**Affiliations:** 1 Laboratory of Medical Microbiology, Vaccine & Infectious Disease Institute, Universiteit Antwerpen, Antwerp, Belgium; 2 MRC Clinical Trials Unit, University College London, London, UK; 3 Bristol Medical School, Bristol, UK; 4 Paediatric Infectious Diseases Research Group, St George’s University of London, London, UK

**Keywords:** Beta-lactams, *Streptococcus pneumoniae*, Emerging serotype, Vaccine escape, Population genomics, Vaccine types, Non-vaccine types

## Abstract

**Background:**

*Streptococcus pneumoniae* is a global cause of community-acquired pneumonia (CAP) and invasive disease in children. The CAP-IT trial (grant No. 13/88/11; https://www.capitstudy.org.uk/) collected nasopharyngeal swabs from children discharged from hospitals with clinically diagnosed CAP, and found no differences in pneumococci susceptibility between higher and lower antibiotic doses and shorter and longer durations of oral amoxicillin treatment. Here, we studied in-depth the genomic epidemiology of pneumococcal (vaccine) serotypes and their antibiotic resistance profiles.

**Methods:**

Three-hundred and ninety pneumococci cultured from 1132 nasopharyngeal swabs from 718 children were whole-genome sequenced (Illumina) and tested for susceptibility to penicillin and amoxicillin. Genome heterogeneity analysis was performed using long-read sequenced isolates (PacBio, *n* = 10) and publicly available sequences.

**Results:**

Among 390 unique pneumococcal isolates, serotypes 15B/C, 11 A, 15 A and 23B1 were most prevalent (*n* = 145, 37.2%). PCV13 serotypes 3, 19A, and 19F were also identified (*n* = 25, 6.4%). STs associated with 19A and 19F demonstrated high genome variability, in contrast to serotype 3 (*n* = 13, 3.3%) that remained highly stable over a 20-year period. Non-susceptibility to penicillin (*n* = 61, 15.6%) and amoxicillin (*n* = 10, 2.6%) was low among the pneumococci analysed here and was independent of treatment dosage and duration. However, all 23B1 isolates (*n* = 27, 6.9%) were penicillin non-susceptible. This serotype was also identified in ST177, which is historically associated with the PCV13 serotype 19F and penicillin susceptibility, indicating a potential capsule-switch event.

**Conclusions:**

Our data suggest that amoxicillin use does not drive pneumococcal serotype prevalence among children in the UK, and prompts consideration of PCVs with additional serotype coverage that are likely to further decrease CAP in this target population. Genotype 23B1 represents the convergence of a non-vaccine genotype with penicillin non-susceptibility and might provide a persistence strategy for ST types historically associated with vaccine serotypes. This highlights the need for continued genomic surveillance.

**Supplementary Information:**

The online version contains supplementary material available at 10.1186/s12866-024-03300-w.

## Introduction

*Streptococcus pneumoniae* (pneumococcus) frequently colonises the nasopharynx of children younger than 5 years. It is also one of the major causes of community-acquired pneumonia (CAP) and invasive disease that require antibiotic treatment. Amoxicillin, a beta-lactam, is widely recommended as first-line treatment for CAP in young children [1]. Resistance selection in adult patients receiving amoxicillin (1 gram, three times daily for seven days) has been shown to be modest and short-lived, potentially due to fitness costs engendered by resistance-conferring mutations in streptococci [2]. Amoxicillin is the WHO recommended treatment for CAP in children, but the optimal dose and duration to maximise clinical efficacy, reduce toxicity and minimise the selection of resistance is unclear.

Pneumococcal infections are also one of the most vaccine preventable infections. Pneumococcal conjugate vaccines (PCVs) have successfully reduced the invasive pneumococcal disease (IPD) burden due to targeted serotypes; however, replacement by non-vaccine serotypes (NVTs) in both nasopharyngeal carriage [3] and invasive disease [4] after PCV implementation has been observed. The observed rise in NVT can be attributed to two main mechanisms: expression of a NVT capsule in a genotypically related PCV serotype (capsular switch) [5], or, more commonly, thriving NVTs replacing VTs in the co-habiting niche [6]. 

Further, *S. pneumoniae* can also naturally activate a competent state that allows uptake of homologous exogenous DNA, and several recombination hotspots have been detected across the genome [7]. These include the penicillin-binding proteins (PBPs), which are the target binding site of the beta-lactams and wherein mutations, especially in PBP1a, PBP2b and PBP2x, result in a lower affinity for this class of antibiotics [6, 7]. Additionally, due to the proximity of *pbp1a* and *pbp2x* genes to the *cps* locus that encodes for the capsular polysaccharide, selective pressure from vaccination or antibiotic treatment might drive both capsule switching and ß-lactam resistance development [7]. 

The CAP-IT trial (Efficacy, safety and impact on antimicrobial resistance of duration and dose of amoxicillin treatment for young children with Community-Acquired Pneumonia (CAP): a randomised controlled trial) enrolled children with CAP that were being discharged from an emergency department, observational unit, or inpatient ward (within 48 h). The study evaluated whether further outpatient treatment with oral amoxicillin at a lower dose was non-inferior to a higher dose, and whether shorter treatment was non-inferior to longer treatment. The trial demonstrated non-inferiority for lower dosage and shorter courses of amoxicillin in terms of antibiotic re-treatment, duration of symptoms, adverse events, and the emergence of phenotypic beta-lactam resistance in *S. pneumoniae* [8]. Here, we performed molecular analysis on unique *S. pneumoniae* isolates obtained from nasopharyngeal swabs (up to two morphologically distinct isolates per sample) collected in the CAP-IT trial prior to and after various doses and duration of amoxicillin treatment to understand the antimicrobial resistance selection, and serotype prevalence in a population with a very high (95%) PCV13 uptake. Further, we explored the genetic features of the persistent VTs detected among our isolates in the context of historical isolates from the UK sharing serotype and ST.

## Methods

### Isolate collection

Isolates were obtained from children recruited into the CAP-IT trial, a randomised, double-blind non-inferiority 2 × 2 factorial trial assessing the efficacy, safety and impact on antimicrobial resistance of dose (35–50 mg kg^−1^ or 70–90 mg kg^−1^) and duration (3 or 7 days) of amoxicillin treatment for children between six months and 6.5 years old, weighing 6–24 kg, from the UK and Ireland with CAP, defined as presence of cough, temperature ≥ 38 °C, and at least one sign of difficult breathing or a focal chest signs (ISRCTN76888927) [9]. Nasopharyngeal swabs (NPS) were collected between 2017 and 2019 and consisted of a baseline (D1) and a follow-up visit at day 28 (D29). Additionally, when unscheduled visits occurred, an additional NPS was collected (USV). Vaccine (PCV13) coverage in this population was 95% and some patients had already received ß-lactams for a period shorter than 48 h prior to randomisation [8]. NPS were stored in skim milk-tryptone-glucose-glycerol (STGG) medium at -20 °C and below within 6 h. For pneumococcal isolation, 50 µL of NPS STGG was plated on streptococcal selective agar COBA (Oxoid, UK) and incubated overnight at 37ºC in 5% CO2. Positive cultures were sub-cultured on blood agar in the same conditions, and species identification was performed by colony morphology, optochin susceptibility test (Sigma-Aldrich, Germany) and bile solubility test (Sigma-Aldrich, Germany). When two different morphologies were observed in the positive culture, both isolates underwent phenotypic identification.

### MIC determination

Penicillin and amoxicillin MICs were determined by broth microdilution, and results were interpreted according to EUCAST guidelines v10.0 for non-meningitis isolates. Penicillin non-susceptibility was considered for MIC > 0.06 mg/L, and resistance for MIC > 2 mg/L. For amoxicillin, non-susceptibility was considered for MIC > 0.5 mg/L, and resistance for MIC > 1 mg/L. *S. pneumoniae* ATCC49619 was used for quality control.

### Whole-genome sequencing

Pneumococcal isolates were inoculated in 4 mL of Todd-Hewitt broth and incubated overnight at 37 °C in 5% CO_2_. DNA was extracted (MasterPure™ Complete DNA and RNA Purification Kit, Epicentre, USA) with few modifications (detailed in Supplementary methods). Libraries were prepared (Nextera XT DNA Library Preparation Kit, Illumina Inc., USA), and sequenced (2 × 250 bp, MiSeq v2 500 cycles kit, Illumina Inc., USA).

In order to delve into the persistence of VTs in our study, a selection of ten VT isolates was long-read sequenced, thus providing a backbone for prediction of recombination events. These included two serotype 3 representatives (ST180), four serotype 19A isolates representing all observed STs (ST199, ST450, ST667 and ST2062), and four serotype 19F isolates representing four different STs (ST179, ST654, ST7024 and ST9972). DNA was extracted using MagAttract HMW DNA kit (Qiagen, Germany), library was prepared using SMRTbell Express Template Prep Kit 2.0 (Pacific Biosciences, USA), and sequencing was performed in the Sequel system (Pacific Biosciences, USA).

### Bioinformatic analysis

Initial WGS analysis was performed using BacPipe v.1.2.6, including read trimming, assembly, annotation, MLST assignment and AMR genes detection [10]. Isolates that were not identified as *S. pneumoniae* by the MLST profiling within BacPipe were re-analysed by rMLST [11], using their assembled contigs, to confirm that they were not pneumococci. Serotyping was performed using PneumoCaT v.1.2.1 [12]. 

Core genome MLST (cgMLST) study-specific scheme was generated using ChewBBACA v.2.1.0 [13] using default settings and *S. pneumoniae* R6 as a reference and visualised with Phyloviz [14]. PBP variants were extracted from the cgMLST scheme and typed according to Li et al. classification [15]. PopPUNK v.2.1.1 [16] was used for genomic epidemiology analysis with default settings, and Global Pneumococcal Sequencing project clusters (GPSC) were assigned according to GPS instructions using version 1.0 (https://www.pneumogen.net/gps/assigningGPSCs.html). PopPUNK results were visualised with Cytoscape v.3.8.0 [17]. 

Parsnp v.1.5.0 [18] was used to align the assembled genomes and the PBP variants with default settings. Phylogenetic trees were generated with RAxML v.8.2.12 from 100 rapid bootstraps with GTRCAT substitution model [19], and genome recombinations were inferred with ClonalFrameML v.1.12 at default settings [20]. All trees were visualised with iTOL v.5.5.1 [21]. 

Long read sequences were subjected to hybrid assembly with their respective short reads using Unicycler v.0.4.8 with default settings [22]. These assemblies were used as a reference to evaluate the recombination history within a serotype or ST. For this, older isolates belonging to the same serotype or ST were retrieved from the pubMLST Penumococcal Genome Library [23] (restricting origin country to United Kingdom, *n* = 229) and Sheppard et al. [24] (*n* = 124) (Table S2). For each serotype and ST, whole genome alignment was performed using Snippy v.4.6.0 with default settings for snippy-multi option (https://github.com/tseemann/snippy), and recombination events were predicted using Gubbins v.2.4.1 with default settings [25]. All alignments were visualised with Phandango v.1.3.0 [26]. Clades for serotype 3 were defined by comparison to sequences classified in Groves et al. [27]. 

## Results

### Isolate characterisation

**Fig. 1:**
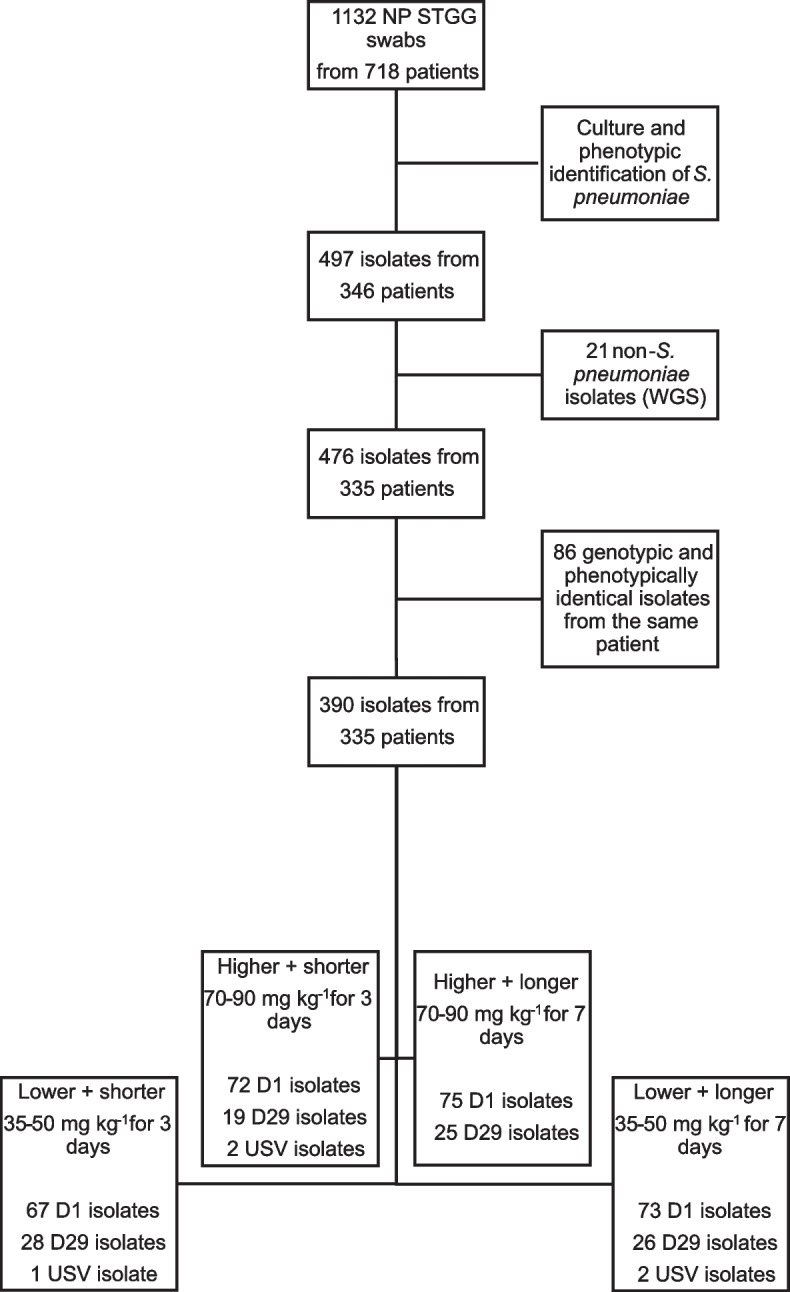
Flow chart depicting isolate collection and filtering process

In total, 497 isolates were obtained from 346 patients. Of these, 21 were identified as non-pneumococci by rMLST (*S. mitis*, *S. parasanguinis*, *S. pseudopneumoniae* and *S. australis*). Furthermore, after MIC determination, serotyping and core genome alignment, 86 isolates were found to be phenotypic and genotypically identical to isolates obtained from the same patient, leaving 390 unique pneumococcal isolates obtained from 335 patients. The isolate distribution across treatment arms and time-points was similar (Fig. 1). It had been previously reported that there were no differences in susceptibility levels between trial arms and between D1 and D29 [8], and serotype distribution across arms was homogenous (Table S3, Supplementary information), thus results described below refer to the entire isolate population.

Overall, 33 different serotypes were detected (Table 1). The most prevalent were all non-PCV13 serotypes, 15B/C (12.3%, 48/390), 11 A (10.3%, 40/390), 15 A (7.7%, 30/390) and 23B1 (6.9%, 27/390) (Table 1). PCV13 serotypes 3 (3.3%, 13/390), 19A (1.8%, 7/390) and 19F (1.3%, 5/390) were detected at a low prevalence, and only one serotype 3 isolate was found in a non-vaccinated patient (Table S1). Only 3.3% (13/390) of the isolates were labelled as non-typeable due to the low (< 30%) coverage of the capsular operon (Table 1), and upon examination of the *cps* locus, nine were classified as Null Capsule Clade (NCC) 2a and the other 4 as NCC2b (Table S1) [28]. No regional clustering of serotypes was found (data not shown).

**Table 1:** Serotype distribution and penicillin and amoxicillin susceptibility among study isolates (*n* = 390). VTs are marked in bold. Shannon diversity index calculated for the STs found in each serotype

Serotype	Number of isolates	Shannon Diversity index (Sequence Types)	Penicillin		Amoxicillin		
			S	I	S	I	R
Total	390		329 (84.4%)	61 (15.6%)	380 (97.4%)	5 (1.3%)	5 (1.3%)
15B/C	48 (12.3%)	1.81	47	1	48		
11A	40 (10.3%)	0.31	37	3	38		2
15A	30 (7.7%)	1.45	21	9	29		1
23B1	27 (6.9%)	1.36		27	27		
21	25 (6.4%)	0.77	25		25		
35F	23 (5.9%)	0.98	22	1	23		
35B	21 (5.4%)	1.71	18	3	19	1	1
10A	18 (4.6%)	1.43	18		18		
23A	18 (4.6%)	0.93	18		18		
**3**	**13 (3.3%)**	**0**	**13**		**13**		
Non-typeable	13 (3.3%)	1.63	6	7	9	3	1
Serogroup 24	11 (2.8%)	0.30	11		11		
22F	10 (2.6%)	0	10		10		
16F	8 (2.1%)	0.38	7	1	8		
33F	8 (2.1%)	1.21	8		8		
7C	7 (1.8%)	0	7		7		
17F	7 (1.8%)	1.00	7		7		
**19A**	**7 (1.8%)**	**1.15**	**6**	**1**	**7**		
9N	6 (1.5%)	0.45	6		6		
12F	6 (1.5%)	0.64	2	4	6		
23B	6 (1.5%)	0.87	6		6		
8	5 (1.3%)	0	5		5		
**19F**	**5 (1.3%)**	**1.61**	**4**	**1**	**4**	**1**	
31	5 (1.3%)	0.50	5		5		
34	5 (1.3%)	1.33	5		5		
38	5 (1.3%)	0	5		5		
6C	4 (1.0%)	0.56	3	1	4		
13	2 (0.5%)	0.69	1	1	2		
35D	2 (0.5%)	0.69	2		2		
6B	1 (0.3%)	0	1		1		
10B	1 (0.3%)	0	1		1		
15F	1 (0.3%)	0		1	1		
20	1 (0.3%)	0	1		1		
37	1 (0.3%)	0	1		1		

**Fig. 2:**
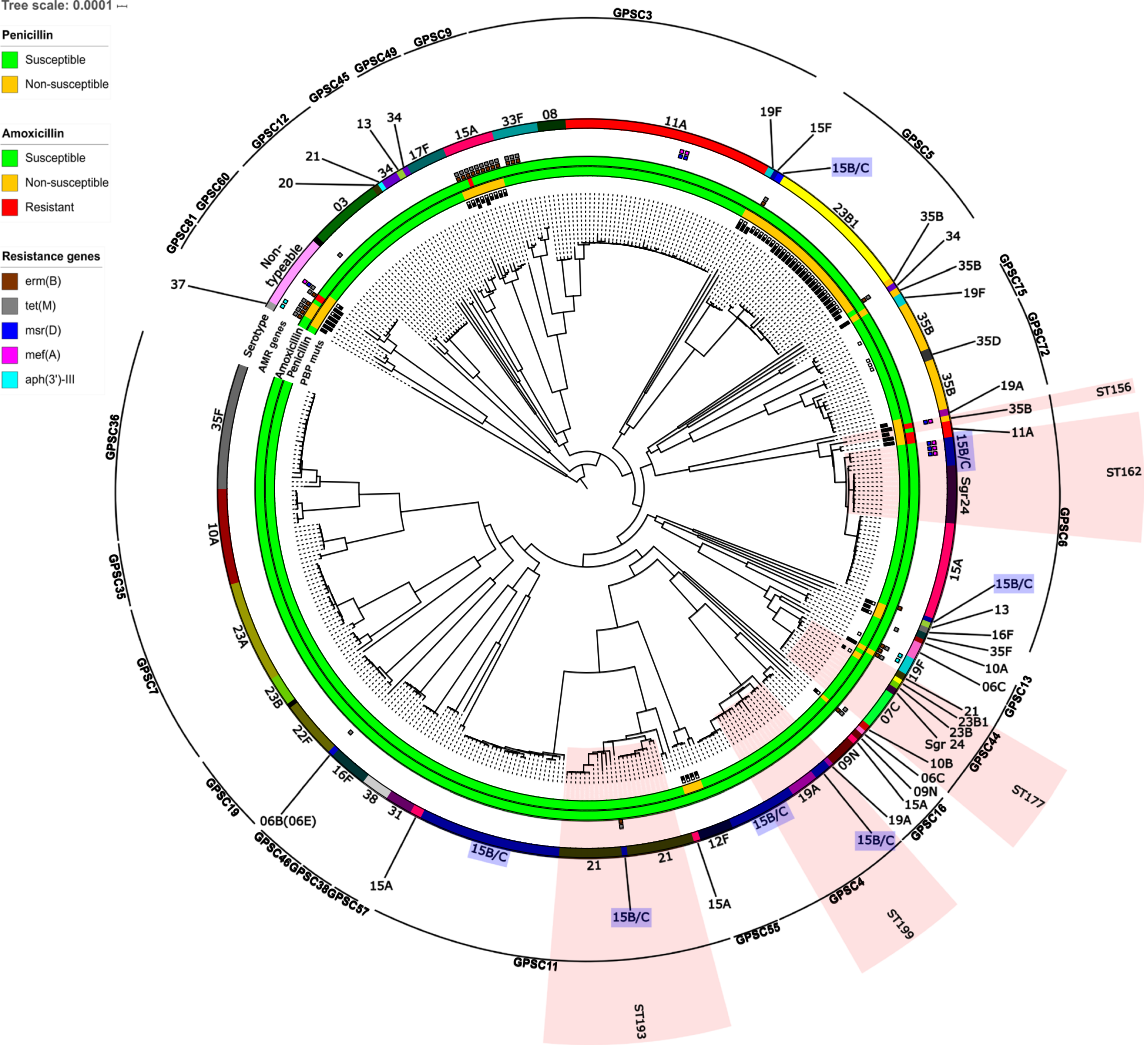
Phylogenetic tree generated from the 390 study isolates’ assemblies. Inner squares represent the presence of amino acid changes in (from inner to outer) penicillin-binding protein (PBP) 1a, PBP2b and PBP2x protein sequences described in the Comprehensive Antibiotic Resistance Database (CARD) to provide non-susceptibility to penicillin. Filled squares represent the presence of all described mutations for PBP1a and PBP2b, and > 4 of the 7 mutations described for PBP2x in CARD. Empty squares depict < 4 PBP2x mutations. Penicillin and amoxicillin susceptibilities are colour-coded (green for susceptible, orange for non-susceptible and red for resistant). Acquired resistance genes are depicted as coloured squares when present in the isolate, potentially conferring resistance to macrolides (brown, blue and purple squares), tetracycline (grey square) and aminoglycosides (light blue square). Outer circles represent serotype of the isolate and Global Pneumococcal Sequence Clusters (only GPSCs presenting > 3 isolates are shown). Finally, pink blocks indicate isolates belonging to the same ST and presenting with different serotypes. Serotype 15B/C was present across 8 STs and is highlighted in blue

Overall, 103 different STs were detected, of which six were novel. Most STs clustered within one serotype (Table S1), however, 5 STs (ST156, ST162, ST177, ST193, ST199) were distributed in 2 or more different serotypes (Fig. 2), indicative of potential capsular switching events. Serotype 15B/C isolates were remarkable in their ST heterogeneity, as nine genomically divergent STs were associated to this serotype (Fig. 2; Table 1, Table S1). A total of 59 local clusters were identified using PopPUNK, and the isolates were classified into 45 GPSCs when using PopPUNK with the GPSC database, indicating some variability within the GPSC clusters. This is a more robust clustering method and related STs can be engulfed into one GPSC, hence the lower number of clusters compared to STs.

**Fig. 3:**
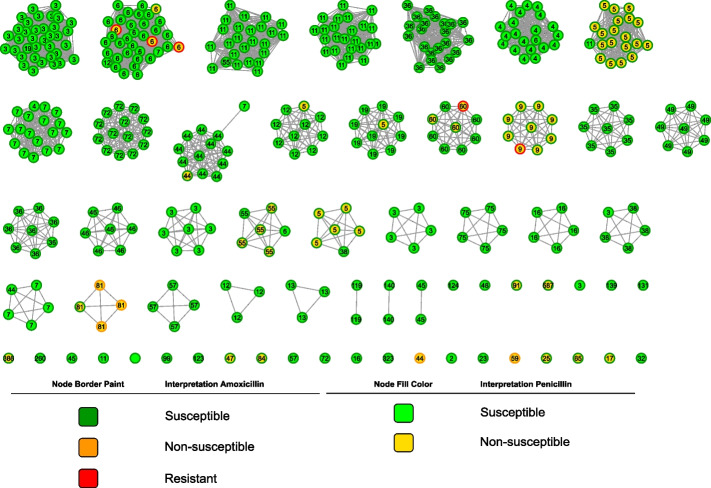
Local popPUNK clustering. Node filling and node border represent penicillin and amoxicillin susceptibility, respectively. Node labels refer to Global Pneumococcal Sequence Clusters

In total, 15.6% (61/390) and 2.6% (10/390) of all isolates were non-susceptible to penicillin and to amoxicillin, respectively. None of the isolates showed resistance to penicillin and 5 were amoxicillin resistant. Penicillin non-susceptible isolates (*n* = 61) were distributed in 13 different serotypes. In four serotypes (11 A, 15 A, 19F, 35B), six isolates (6.3%, 6/96) were penicillin and amoxicillin non-susceptible (Table 1). Additionally, non-typeable isolates presented a high prevalence of penicillin (7/13) and amoxicillin (4/13) non-susceptibility (Table 1). All genotype 23B1 (*n* = 27) and serotype 15 F (*n* = 1) isolates were found to be non-susceptible to penicillin, and susceptible to amoxicillin (Table 1). Seventeen GPSCs showed penicillin non-susceptibility, and of these six (6, 44, 60, 9, 81, 59) also showed amoxicillin non-susceptible isolates (Fig. 3). Remarkably, all isolates in GPSCs 5 (genotype 23B1) and 9 (serotype 15 A) were penicillin non-susceptible.

**Fig. 4:**
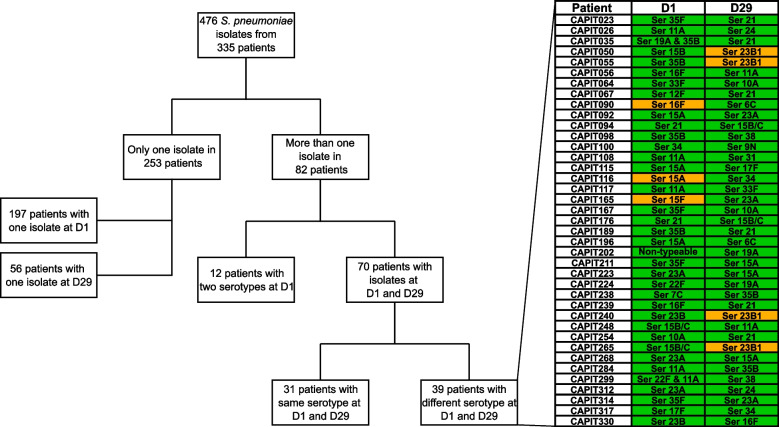
Flow chart depicting isolate distribution within patients across samples, and table showing in detail the serotypes and penicillin non-susceptibility observed in patients where two different serotypes were found at D1 and D29. Green denotes penicillin susceptibility and orange penicillin non-susceptibility

From the 39 patients presenting different serotypes at D1 and D29, only in four instances a penicillin susceptible isolate was replaced by a penicillin non-susceptible one at D29 after antibiotic treatment, and, remarkably, in all these instances the replacing serotype was 23B1 (Fig. 4). In three other instances, a penicillin non-susceptible isolate was replaced by a susceptible one, while in the remaining 32 cases, both unique isolates at D1 and D29 were susceptible to penicillin (Fig. 4). None of the replacing isolates was resistant to amoxicillin.

In total, 73 acquired resistance genes were detected in 38 isolates (9.7%, 38/390), conferring resistance primarily to macrolides and tetracycline, although susceptibility testing was not performed for these antibiotics (Table S1). The most prevalent genes were *tet(M)* (*n* = 28) and *erm(B)* (*n* = 25), and were mostly co-integrated in the chromosome (5.9%, 23/390). In 21 isolates, these genes were flanked by a Tn916 family transposon (Table S1). Similarly, *mef(A)* and *msr(D)* were present together in 7 isolates. Only 4/390 (1%) of the isolates carried an aminoglycoside resistance gene (*aph (3’)-III*), which was always found to be integrated along with *erm(B)* (Fig. 2).

### Geno-pheno-type correlation

**Fig. 5:**
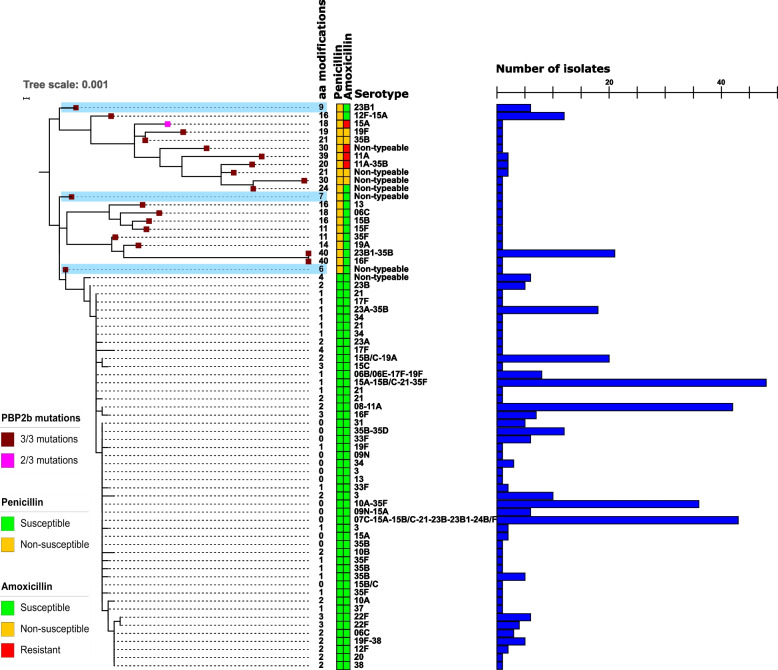
Phylogenetic tree generated from penicillin-binding protein 2b amino acid sequences. Coloured squares represent the presence of mutations previously described to confer an increase in MIC. Number of total amino acid modifications, penicillin and amoxicillin susceptibility, serotype, and number of isolates are also depicted. Variants highlighted in blue present a low number of overall amino acid modification while containing the three key modifications described in the CARD database to reduce susceptibility to beta-lactams

From the generated cgMLST scheme, sequences from PBP amino acid variants were extracted and analysed from the study population, totalling 65 PBP1a, 70 PBP2b and 78 PBP2x variants, respectively. These alleles presented an average of 19 (2-108/720, 97.3% identity), 7 (0–40/681, 99.0% identity) and 19 (0–78/751, 97.5% identity) amino acid modifications compared to the reference strain R6, respectively (Figs. 5 and S2).

The presence of specific PBP mutations, as described in the CARD database (derived from Stanhope et al.) [29], explained penicillin non-susceptibility, especially in the case of PBP2b (Fig. 5). Upon comparison to non-*S. pneumoniae* isolates found in our collection, the PBP variants conferring beta-lactam non-susceptibility were found to be genetically similar to those in *S. mitis* and *S. pseudopneumoniae* (data not shown).

All identified PBP variants were subjected to protein-based phylogenetic analysis, which showed clustering of mutated variants and a correlation between key mutations and overall number of changes in comparison to the reference sequence, that is, the variants presenting the key mutations described in the CARD database contained a higher number of total amino acid modifications compared to the reference, indicating that these variants arose from recombination (i.e., mosaic genes), and the total number of mutations found in the three PBPs correlated with increasingly higher MICs to both penicillin and amoxicillin (Supplementary information, Results).

All identified PBP2b variants clustered into three clades of which the penicillin non-susceptible isolates clustered exclusively in two clades (Fig. 5). Of note, 3 PBP2b variants (*n* = 8) were unique in that they harboured 3 predicted amino acid changes that are linked to beta-lactam non-susceptibility (T445A, E475G, T488A), however, the rest of the gene was highly conserved (6, 7 and 9 amino acid modifications, i.e. >98.5% identity), indicating that these variants were the result of *de novo* mutations instead of recombination events and mosaicism as observed for other PBPs.

We found 64 different combinations of PBP variants (PBP types) in our collection, of which 38 had already been described and associated to MIC values that match the ones we obtained [15]. Twenty-four novel variants were observed, of which 18 were found in penicillin non-susceptible isolates. In general, PBP type was consistent within STs and variation within ST 156, 162, 177 and 199 was explained by serotype (Table S1).

### Genomic analysis of persistent vaccine types

#### Low genomic heterogeneity in serotype 3 and predominance of clade Ia

**Fig. 6:**
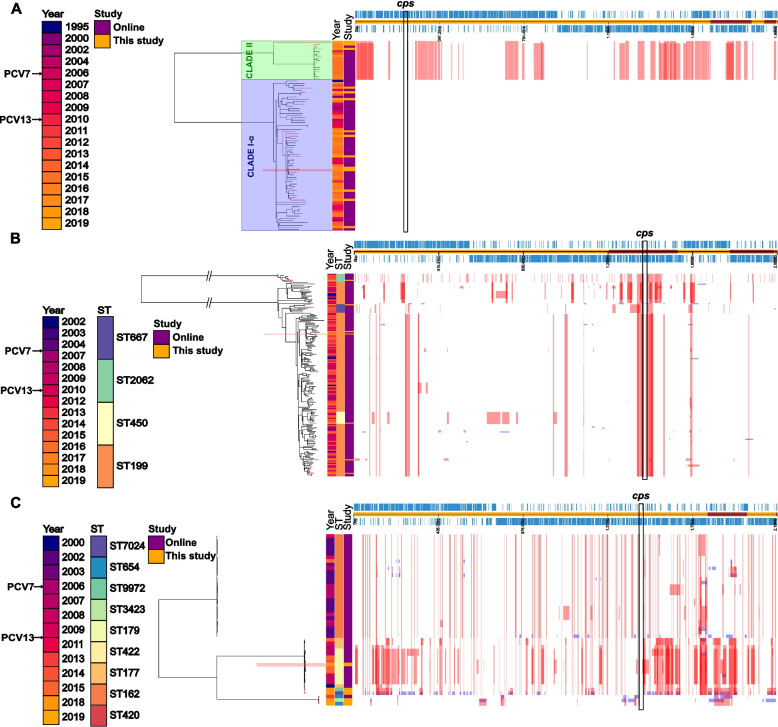
Phylogenetic trees generated from whole genome alignments of serotype 3 (**A**), serotype 19A (**B**) and serotype 19F (**C**) isolates derived from this study (orange shading) and older isolates from the same serotypes. References used were CAPIT119_D1-1, CAPIT226_D1-1 and CAPIT214_D1-1 (marked in red shading), respectively. In red, are shown the large genomic changes predicted to have occurred earlier in evolution, thus being present in a cluster of isolates. In blue, genomic changes predicted only in the branch leaves, that is, in only one isolate. The position of the cps locus in the core genome is indicated with a black rectangle. For serotype 3, the clade to which the isolates belong is also depicted. All serotype 3 isolates belonged to sequence type 180

Genome stability varied greatly between serotypes, indicated by the within-serotype ST diversity (Table 1). All serotype 3 isolates belonged to ST180, and genomic differences were only observed between clades, suggesting that a serotype 3 clade is a temporally stable unit (Fig. 6A). In our study collection, 10/13 (76.9%) of serotype 3 isolates clustered in clade Ia and only three isolates belonged to clade II, which contained 14 non-study UK isolates from 2010 onwards (Fig. 6A). Additionally, no genomic changes were observed surrounding the *cps* locus (Figure S3A).

### High genomic heterogeneity in serotypes 19A and 19F

Serotypes 19A and 19F presented a higher genomic complexity, even within the same ST. Serotype 19 A isolates belonging to ST2062 formed a separate clade when compared to the rest of serotype 19A isolates, which were clustered in three sub-clades (Fig. 6B). The biggest sub-clade was composed of isolates belonging to ST199 and ST450, while the two smaller sub-clades were composed of ST667 and ST199 isolates (Fig. 6B). Genomic differences surrounding the *cps* locus in ST199 and ST450 were more pronounced than in ST667, wherein the region upstream of the *cps* locus was less divergent compared to the other STs, although the region between *pbp1A* and *dexB* was highly variable in all three STs (Figure S3B).

Finally, serotype 19F isolates presented higher within-ST genome variability (Fig. 6C). Isolates belonging to ST162, ST420 and ST422 were not detected after 2009, except for one ST162 isolate in 2015. Overall, 19F isolated from 2006 onwards were observed to have undergone more genomic changes (Figure S3C).

### Within ST serotype variability might facilitate vaccine escape

**Fig. 7:**
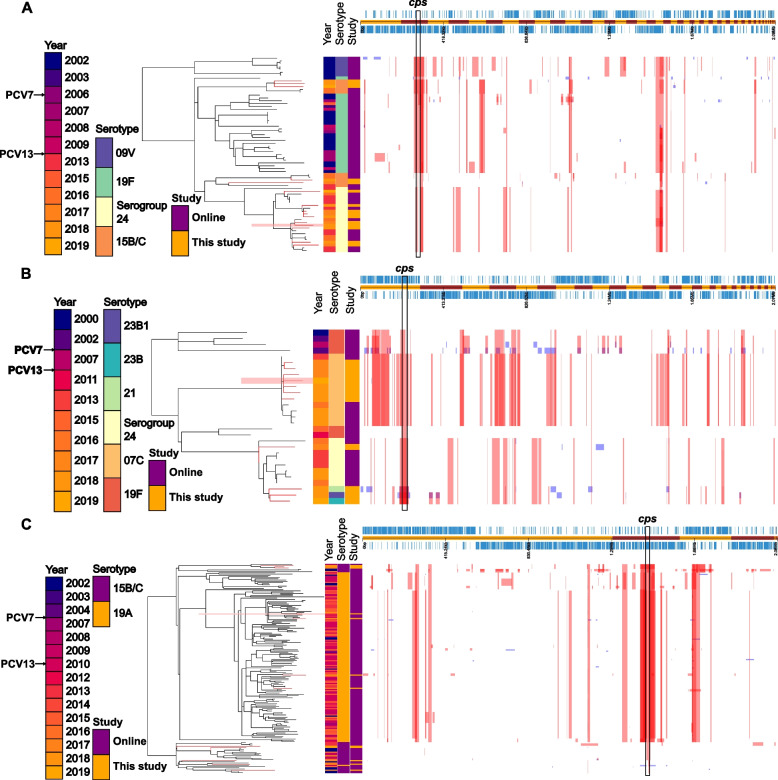
Phylogenetic trees generated from whole genome alignments of sequence type (ST) 162 (**A**), ST177 (**B**) and ST199 (**C**) isolates derived from this study (orange shading) and older isolates from the same serotypes. References used were CAPIT086_D1-1, CAPIT292_D1-1 and CAPIT226_D1-1 (marked in red shading), respectively. In red are shown the large genomic changes predicted to have occurred earlier in evolution, thus being present in a cluster of isolates. In blue, genomic changes predicted only in the branch leaves, that is, in only one isolate. The position of the cps locus in the core genome is indicated with a black rectangle

In order to observe possible capsular switch events leading to vaccine escape, isolates belonging to ST162, ST177 and ST199 were also studied, as these STs were detected in our collection to be associated with several different serotypes. In the case of ST162, isolates presenting serotype 9V were not detected after PCV7 implementation, and were very divergent from the rest of isolates (Fig. 7A). However, isolates presenting serotype 19F could still be detected after PCV7, but in a lower proportion, and only one isolate was observed after PCV13 implementation (Fig. 7A). Isolates presenting serogroups 15 and 24 only started to be detected after PCV13 implementation, and they were more related to serotype 19F isolates than to 9V, but presenting lower genomic divergence, especially around the cps locus (Figure S4A).

ST177 was less represented in the pubMLST database, although 3.7% (11/390) belonged to this ST in our collection. We show here a serotype divergence in ST177: two clades were clearly differentiated, one including serotype 19F isolates pre-PCV7, and one including isolates presenting different serotypes and only detected after PCV13 introduction (Fig. 7B). Remarkably, three ST177 isolates from our collection that were genomically very similar presented with serotypes 21, 23B and 23B1 (Fig. 7B). These showed slight divergence from the closely-related serogroup 24 isolates, which might indicate a higher potential for these isolates to switch capsule, as can be observed by the genomic variability surrounding the *cps* locus (Figure S4B). ST177 presenting serotype 7C were only detected after 2015 and presented a similar additional genomic variability compared to the genome structure of serotype 19F isolates before PCV13 implementation (Fig. 7B).

Finally, isolates belonging to ST199 only presented serotypes 15B/C or 19A. In general, isolates clustered according to their serotype (Fig. 7C). Remarkably, one serotype 19A isolate from 2010 clustered together with serotype 15B/C isolates, and the biggest observed divergence was only surrounding the *cps* locus, indicating a potential capsule switch event to 19A before the PCV13 implementation (Figure S4C).

## Discussion

*Streptococcus pneumoniae* continues to be one of the main pathogens causing pneumonia in the paediatric population. Here, we studied the pneumococcal population carried in the nasopharynx of children aged between 0.5 and 6.5 years attending hospital with CAP in the UK and Ireland, and treated with varying doses and durations of amoxicillin. Children were recruited between February 2017 and April 2019 from 29 different hospitals in the UK and Ireland and 95% of them had followed routine vaccination. None of the amoxicillin regimens tested here resulted in an increase in beta-lactam resistance among the isolated pneumococci, although sampling at D29 rather than at the end of therapy might have underestimated resistance emergence. These results corroborate our previous study in adults where a small but significant increase in amoxicillin resistance was observed at D8 (within 24 h of end of amoxicillin therapy) compared to the placebo, but was not sustained by the D28 sampling [2]. Additionally, a large study of 4,000 IPD cases observed that time elapsed since the last penicillin treatment is significantly associated with antibiotic non-susceptibility, with a high drop in probability to find non-susceptible isolates after the first month post-therapy [30]. 

Nonetheless, antimicrobial use has been one of the major driving forces underlying the evolution of antimicrobial resistance in the pneumococcus. Analysis of 31,000 pneumococcal strains from Finland showed a strong correlation between macrolide resistance and previous macrolide or azithromycin use, as well as between previous beta-lactam or cephalosporin treatment and penicillin resistance [31]. Additionally, a comparison of high-dose, short-course amoxicillin treatment to a standard, low-dose, long-course regimen showed a lower risk of carriage of non-susceptible pneumococci at day 28 in the high-dose, short-course arm [32]. With the introduction of PCV7, which included five serotypes strongly associated with antimicrobial resistance (6B, 9V, 14, 19F and 23F) [33], reduction in carriage of these serotypes consequentially impacted antimicrobial resistance incidence amongst pneumococci. Another consequence of PCV vaccination has been the phenomenon of ‘serotype replacement’ by NVTs.

In our study, vaccine serotypes covered by PCV13 (including 3, 19A and 19F), were identified at a low prevalence (6.4%), although higher than the 1% reported in 2015-16 for children < 5 years old [34], and all but one serotype 3 isolate were found in vaccinated patients. This might be explained by the disease status of the children, as PCV13 serotypes are known to have a higher disease-causing potential, evidenced by the decrease in IPD after PCV introduction [35]. 

Isolates belonging to serotype 19F (included in PCV7, 10 and 13, as well as in PPV23) were detected (1.3%, 5/497), which were not found in a recent carriage study in healthy children and their household contacts [34], but accounted for 2.4% of IPD cases in 2016/17 [4]. Similar findings have been reported in Australia [36] and the United States [37], where 19F was the most prevalent PCV7 serotype causing IPD. The within-ST serotype 19F high variability in our analysis, especially ST177, might underlie their persistence. In support of our hypothesis, 19F isolates belonging to several historical STs (ST162, ST420, ST422) were not detected in our collection, and seem to have been effectively targeted by vaccination, as they have not been detected in the UK since 2010 (except for one ST162 isolate in 2015). On the other hand, ST162 isolates presenting NVTs (15B/C and serogroup 24) have been observed pre-PCV and became more prevalent after PCV implementation, probably driven by ST/lineage expansion rather than by capsule switching events [24]. 

Post-PCV13 persistence in carriage of serotypes 3 and 19A strains has been described by many studies [4, 5, 24, 37]. Serotype 3 isolates exclusively belonged to ST180 and GPSC12 in our collection, which suggests that this VT has not needed capsular switching to escape vaccine pressure. Indeed, when compared to older serotype 3 isolates from the UK, very few genetic differences were detected, and only between clades, indicating very high within-clade genome stability [27, 38]. Remarkably, none of these differences were found close to the *cps* locus, a known hotspot of recombination, further emphasizing the genomic stability of this serotype.

The potential lack of protection from PCV13 against serotype 3 colonisation and/or invasion has been suggested to be related to the production mechanism of the serotype 3 capsule, which follows the synthase-dependent pathway, allowing capsule release from the peptidoglycan, and affording immune protection for this serotype [39]. Additionally, the clades differentially express polymorphic pneumococcal protein antigens that can impact carriage duration, transmission and invasiveness [38]. 

Serotype 19A, on the other hand, presented a higher genetic diversity, and was found to belong to four STs and two GPSCs. Post-PCV7, this serotype has been associated with high-level beta-lactam resistance as a result of capsular switching and recombination with resistant PCV7 VTs [6]. Additionally, capsule switch events have been described involving serotypes 19A and 15B/C belonging to ST199 [40], which was the most common ST among our serotype 19A isolates. In our collection, one serotype 19A was found within 15B/C in the ST199 cluster, and conversely, a 15B/C cluster only detected from 2010 onwards was closely related to a 19A cluster that was not detected after 2013. Further, sharing of a GPSC by a VT and NVT (19A and 15B/C) highlight the increased potential of such NVTs to replace the closely related VTs [41]. 

A remarkable finding was that 100% of the 23B1 genotype isolates (*n* = 27) were non-susceptible to penicillin, and in four instances, this genotype replaced a susceptible serotype after antibiotic treatment. This genotype has only been detected from 2005 onwards and was characterised as a genotype of 23B, as it produces the same polysaccharide. However, the *cps* locus at the 3’ end shows genetic similarity to that of 19A [42]. Thus, differentiation of the genotype can only be performed by WGS, which likely explains the lack of isolates prior to 2005. Most 23B1 genotype isolates belonged to ST1349, ST2372 and ST1373, as previously reported [42], and all but one were assigned to GPSC5, being the only representatives of this lineage in our collection. This lineage is among the ten most prevalent globally, and has been described to present a high resistance prevalence to penicillin and other classes of antibiotics, although this lineage has been associated with other serotypes in different countries, such as 35B/D in South Africa or 19A in Israel [5]. Given the increase in 23B1 prevalence and the similarity to other successful serotypes around the world, this chimeric genoype potentially presents a fitness advantage compared to serotype 23B, while conserving penicillin non-susceptibility, and might present a route for vaccine escape via serotype switching, warranting further investigation.

A major limitation of our study was the use of culture to isolate pneumococci, as this approach precludes the study of the entire pneumococcal population in a sample due to similar colony morphology between serotypes or STs. Despite being able in some cases to isolate multiple serotypes from the same sample, this was not always possible, and this might have led to an underestimation of the serotype prevalence, and potentially of antibiotic resistance, although multiserotype carriage was not expected to be high in this population. Furthermore, while use of WGS for serotyping is widespread, some errors can arise (e.g. low coverage of the capsular locus), which were manually curated. Additionally, the isolates were obtained from the nasopharynx of patients with CAP, and while these cannot be definitively considered as the aetiological agents, we also could not classify these as commensals given the disease status of the population at baseline. Finally, lack of healthy controls precluded any estimation of paediatric pneumococcal carriage rates and serotypes.

To conclude, our data show a lack of persistence of amoxicillin non-susceptibility one month after treatment, which was also reflected in the low levels of amoxicillin non-susceptibility in the studied pneumococci and is in agreement with previous studies, suggesting that amoxicillin consumption is not a major driver of pneumococcal serotype dynamics among children in the UK, and leaving host immunity due to vaccination as the main driver of colonization in this population. Thus, considering PCVs as the main driver of these dynamics, our results indicate that the introduction of PCVs with wider serotype coverage would result in further decrease in invasive disease caused by NVTs included in these new formulations that have been observed to effectively replace PCV13-targeted serotypes. We also demonstrate the different genomic features of pneumococcal serotypes that persist, albeit at a low prevalence, despite being included in PCV13. Finally, we highlight the emergence of 23B1, a non-vaccine, penicillin-non-susceptible genotype harbouring a 23B/19A chimeric capsular polysaccharide locus that might provide a persistence strategy for vaccine serotypes and exemplifies the need for continued genomic surveillance.

### Supplementary Information

**Additional File 1. Table S1**

**Additional File 2. Table S2**

**Additional File 3. Supplementary information, supplementary figures and Table S3**

## Data Availability

The datasets generated and analysed during the current study are available at ENA under bioproject number PRJEB55546 and at NCBI with bioproject number PRJNA798685.
