# Supplementary material for: High-resolution genomics identifies pneumococcal diversity and persistence of vaccine types in children with community-acquired pneumonia in the UK and Ireland
Source: BMC Microbiol. 2024 Apr 27;24:146. doi: 10.1186/s12866-024-03300-w (PMC11055344; doi:10.1186/s12866-024-03300-w)
Supplement: Supplementary file 3 — Additional File 3. Supplementary information, supplementary figures and Table S3 [file 12866_2024_3300_MOESM3_ESM.docx]

# **Supplementary Information**

## **Isolation and identification of pneumococci**

50 μL of NPS STGG was plated on streptococcal selective agar COBA (Oxoid, UK) and incubated overnight at 37ºC in 5% CO2. Positive cultures were sub-cultured on blood agar in the same conditions, and species identification was performed by colony morphology, optochin susceptibility test (Sigma-Aldrich, Germany) and bile solubility test (Sigma-Aldrich, Germany). When two different morphologies were observed in the positive culture, both isolates underwent sub-culture and phenotypic identification.

## **Whole-genome sequencing**

Pneumococcal isolates were inoculated in 4 mL of Todd-Hewitt broth and incubated overnight at 37°C in 5% CO_2_. DNA was extracted using MasterPure™ Complete DNA and RNA Purification Kit (Epicentre, USA) according to manufacturer’s instructions with few modifications. Briefly, 1 mL of the overnight culture was pelleted and resuspended in 150 µL TE buffer. Ready-to-Lyse lysozyme (Epicentre, USA) and mutanolysin (Sigma-Aldrich, Germany) 5000 U ml^-1^ were added, and the solution was incubated at 37°C for 30 minutes. 150 µL of Tissue and Cell Lysis Solution containing Proteinase K (50 µg µl^-1^) was added, after which the manufacturer’s protocol was followed.

After DNA extraction, an additional cleaning and concentration step was performed using DNA Clean & Concentrator™-10 (Zymo Research, USA) according to the manufacturer’s instructions. DNA concentration was determined using Qubit Fluorometer 3.0 (Thermo Fischer Scientific, USA).

## **CAP-IT trial**

One of the secondary endpoints of the trial was phenotypic penicillin non-susceptibility or resistance in nasopharyngeal *S. pneumoniae*. Penicillin non-susceptibility in patients with a culture positive for *S. pneumoniae* was 17% at baseline and 16% 28 days later, and no significant differences were observed between the different arms of the trial, indicating that the treatment had no effect in pneumococcal non-susceptibility levels, or this effect had been lost before the follow-up visit. Finally, serotypes were evenly distributed across the different arms (Table S3). For all these reasons, it was decided to study the overall isolate population instead of focusing on differences between the trial arms.

## **Selection of isolates**

In total, 497 isolates were obtained from 346 patients. Of these, 21 were identified as non-pneumococci. By rMLST. Species identified included *S. mitis*, *S. parasanguinis*, *S. pseudopneumoniae* and *S. australis*. Furthermore, when after MIC determination, serotyping and core genome alignment, 86 isolates were found to be phenotypic and genotypically identical to isolates obtained from the same patient, leaving 390 unique pneumococcal isolates obtained from 335 patients.

## **Acquired resistance genes**

The most prevalent genes were *tet(M)* (n=28) and *erm(B)* (n=25), and were mostly co-integrated in the chromosome (5·9%, 23/390). In 21 isolates, these genes were flanked by a Tn916 family transposon (Table S1). Similarly, *mef(A)* and *msr(D)* were present together in 7 isolates. Only 4/390 (1%) of the isolates carried an aminoglycoside resistance gene (*aph (3’)-III*), which was always found to be integrated along with *erm(B)* (Figure 2).

## **Core genome MLST (cgMLST)**

cgMLST analysis yielded a total of 1391 core genes and an average distance between isolates of 1161·9 (3-1318). As observed in the phylogenetic tree, some serotypes, such as 15B/C or 35B, were spread across the tree, but, in general, isolates presenting the same serotype clustered together (Figure S1A). On the other hand, clustering by GPSC was more robust, and all isolates assigned to the same GPSC clustered closely (Figure S1B).

## **Penicillin-binding proteins**

Based on the phylogenetic analysis, PBP1a variants formed two distinct clades, the first one containing four divergent variants corresponding to non-typeable isolates, and the second one presenting a differentiated sub-clade containing the rest of divergent variants, where the distances increased along with the increase in the number of amino acid modifications (Figure S3A). Interestingly, PBP1a variants from non-typeable isolates consistently showed more divergence compared to the reference protein sequence (7.8-15% divergence, 56-108 amino acid modifications).

In PBP2x variants, no clear division was identified. However, it can be observed that a higher number of total modifications correlates with a higher accumulation of the seven mutations previously described to confer resistance (Figure S2B).

Usually, when a PBP variant presented a high number of amino acid changes, it was accompanied by other PBP variants also with a high number of changes, further reflecting the recombination origin of these changes (Table S1).

## **Within ST recombinations might facilitate vaccine escape**

In order to observe possible capsular switch events leading to vaccine escape, isolates belonging to STs 162, 177 and 199 were also studied, as these STs were detected in our collection to be composed of several different serotypes. In the case of ST162, isolates presenting serotype 9V were not detected after PCV7 implementation, and were very divergent from the rest of isolates (Figure S4A). However, isolates presenting serotype 19F could still be detected after PCV7, but in a lower proportion, and only one isolate was observed after PCV13 implementation (Figure S4A). Isolates presenting serogroups 15 and 24 only started to be detected after PCV13 implementation, and they were more related to serotype 19F isolates than to 9V, but presenting lower genomic divergence, especially around the *cps* locus (Figure S5A).

ST177 was less represented in the pubMLST database, although 3.7% (11/390) belonged to this ST in our collection. Two clades are clearly differentiated, one including serotype 19F isolates pre-PCV7, and one including isolates presenting different serotypes and only detected after PCV13 introduction (Figure S4B). Remarkably, three isolates from our collection presenting serotypes 21, 23B and 23B1 were clustered together and were genotypically very similar, presenting slight divergence from closely related serogroup 24 isolates, which might indicate a higher potential for these isolates to switch capsule, as can be observed in the recombination event prediction surrounding the *cps* locus (Figure S5B). Isolates presenting serotype 7C were only detected after 2015 and presented a similar recombination pattern to that of serotype 19F isolates within the clade, although the region surrounding the *cps* locus showed divergence (Figure S4B).

Finally, isolates belonging to ST199 only presented serotypes 15B/C or 19A. In general, isolates clustered according to their serotype (Figure S4C). Remarkably, one serotype 19A isolate from 2010 clustered together with serotype 15B/C isolates, and the biggest observed divergence was only surrounding the *cps* locus, indicating a potential capsule switch event between these isolates (Figure S5C). Additionally, a small sub-cluster of serotype 15B/C isolates detected only after 2010 was found to present a recombination pattern different from the rest of 15B/C isolates, as observed previously with ST199 serotype 19A isolates (Figure S4C).

# **Supplementary Figures**





**Figure S1.** Tree generated form core-genome multi-locus sequence typing analysis. Nodes are coloured according to (A) serotype and (B) Global Pneumococcal Sequence Cluster. Due to restrictions from Phyloviz, only the 20 most prevalent serotypes and GPSCs are represented.





**Figure S2.** Phylogenetic trees generated from penicillin-binding protein (PBP) 1a (A) and PBP2x (B) amino acid sequences. Coloured squares represent the presence of mutations previously described to confer non-susceptibility. Number of total amino acid modifications, penicillin and amoxicillin susceptibility, serotype, and number of isolates are also depicted.





**Figure S3.** Phylogenetic trees generated from the *cps* locus region of serotype 3 (A), serotype 19A (B) and serotype 19F (C) isolates derived from this study (orange shading) and older isolates from the same serotypes. References used were CAPIT119_D1-1, CAPIT226_D1-1 and CAPIT214_D1-1 (marked in red shading), respectively. In red are shown the recombination events predicted to have occurred earlier in evolution, thus being present in a cluster of isolates. In blue, recombination events predicted only in the branch leaves, that is, in only one isolate. The position of the *cps* locus is indicated with green boxes, and *pbp* genes with red boxes. For serotype 3, the clade to which the isolates belong is also depicted. All serotype 3 isolates belonged to sequence type 180.





**Figure S4.** Phylogenetic trees generated from the *cps* locus region of sequence type (ST) 162 (A), ST177 (B) and ST199 (C) isolates derived from this study (orange shading) and older isolates from the same serotypes. References used were CAPIT086_D1-1, CAPIT292_D1-1 and CAPIT226_D1-1 (marked in red shading), respectively. In red are shown the recombination events predicted to have occurred earlier in evolution, thus being present in a cluster of isolates. In blue, recombination events predicted only in the branch leaves, that is, in only one isolate. The position of the *cps* locus is indicated with green boxes, and *pbp* genes with red boxes.

# **Supplementary Tables**

**Table S3.** Serotype distribution across the different arms of the trial.

| **Arm** | **15B/C** | **11A** | **15A** | **23B1** | **21** | **35F** | **35B** | **23A** | **10A** | **03** | **Sg 24** | **22F** | **NT** | **33F** | **16F** | **17F** | **07C** |
| --- | --- | --- | --- | --- | --- | --- | --- | --- | --- | --- | --- | --- | --- | --- | --- | --- | --- |
| Higher + longer | 14 | 8 | 11 | 4 | 5 | 6 | 7 | 5 | 3 | 3 | 4 | 6 | 3 | 2 | 3 | 3 | 1 |
| Higher + shorter | 14 | 12 | 6 | 9 | 7 | 4 | 3 | 3 | 5 | 4 | 3 | 1 | 4 |  |  |  | 5 |
| Lower + longer | 8 | 10 | 7 | 6 | 7 | 8 | 7 | 4 | 6 | 2 | 2 | 2 | 1 | 2 | 3 | 1 | 1 |
| Lower + shorter | 12 | 10 | 6 | 8 | 6 | 5 | 4 | 6 | 4 | 4 | 2 | 1 | 5 | 4 | 2 | 3 |  |
| **Total** | **48** | **40** | **30** | **27** | **25** | **23** | **21** | **18** | **18** | **13** | **11** | **10** | **13** | **8** | **8** | **7** | **7** |
|  |  |  |  |  |  |  |  |  |  |  |  |  |  |  |  |  |  |
|  |  |  |  |  |  |  |  |  |  |  |  |  |  |  |  |  |  |
| **Arm** | **19A** | **23B** | **12F** | **09N** | **34** | **38** | **31** | **08** | **19F** | **06C** | **13** | **35D** | **20** | **06E** | **15F** | **10B** | **37** |
| Higher + longer | 1 | 2 |  | 1 | 2 | 2 |  |  |  | 2 |  | 1 |  | 1 |  |  |  |
| Higher + shorter | 2 | 3 | 2 |  |  | 1 | 1 | 1 | 2 |  |  |  |  |  |  | 1 |  |
| Lower + longer |  | 1 | 4 | 3 | 2 | 2 | 3 | 3 | 2 |  | 1 | 1 | 1 |  |  |  | 1 |
| Lower + shorter | 4 |  |  | 2 | 1 |  | 1 | 1 | 1 | 2 | 1 |  |  |  | 1 |  |  |
| **Total** | **7** | **6** | **6** | **6** | **5** | **5** | **5** | **5** | **5** | **4** | **2** | **2** | **1** | **1** | **1** | **1** | **1** |
